# *Myodes rufocanus* Cataract Identification and Transcriptome Analysis

**DOI:** 10.3390/genes17050495

**Published:** 2026-04-22

**Authors:** Mingzhe Wang, Qiuyun Zhou, Shengnan Han, Yulu Geng, Xianfeng Yu, Fushi Quan

**Affiliations:** Department of Laboratory Animals, College of Animal Sciences, Jilin University, 5333 Xi’an Road, Changchun 130062, China; 15526660925@163.com (M.W.); zhouqy24@malls.jlu.edu.cn (Q.Z.); hansn1117@163.com (S.H.); gengy11998@163.com (Y.G.); xianfeng79@jlu.edu.cn (X.Y.)

**Keywords:** *Myodes rufocanus*, cataract, animal model, pathological histology, transcriptome

## Abstract

Background: Cataract is a progressive lens opacity. According to the World Health Organization, about 45 million people in the world are blind, with about half of these cases attributable to cataracts. Due to the complexity of cataract disease, current research on cataracts is far from sufficient, so it is especially important to understand the development process and the pathogenic factors of cataracts. *Myodes rufocanus* (*M. rufocanus*) is an animal of the *M. rufocanus* of the hamster family Volinae. In developing *M. rufocanus*, we found an individual of *M. rufocanus* with a congenital cataract phenotype. We confirmed the symptoms of cataract under natural light and using a slit lamp. Methods: Therefore, we analyzed the mechanism of congenital cataract in *M. rufocanus* from the aspects of pathological histology, physiology and biochemistry, and gene level, aiming to explore the feasibility of its development into an animal model of cataract. Cataract is a progressive lens opacity and a leading cause of visual impairment. Understanding its pathogenesis requires appropriate animal models. In a laboratory-bred colony of *M. rufocanus*, we identified individuals with a spontaneous congenital cataract phenotype, confirmed by gross observation and slit lamp examination. To characterize this phenotype and explore its potential as an animal model, we performed pathological, physiological, biochemical, and transcriptomic analyses using three cataract-affected and three normal age-matched male individuals (8 weeks old per group). Results: Blood tests revealed significantly lower white blood cell and lymphocyte counts in the cataract group, while blood glucose and other biochemical parameters showed no significant differences. Histologically, cataractous lenses exhibited eosinophilic aggregation in the nuclear region with disorganized fiber cells. Transcriptome analysis identified 6544 differentially expressed genes, including downregulation of crystallin genes (CRYBB2, CRYBA4, CRYGS) known to be associated with congenital cataract. KEGG pathway enrichment analysis highlighted retinol metabolism, tyrosine metabolism, and cytochrome P450-related pathways. RT-qPCR confirmed reduced CRYBB2 expression in cataractous eyes. Conclusions: This study provides the first transcriptome dataset for *M. rufocanus* ocular tissues and offers preliminary evidence that this naturally occurring cataract phenotype may serve as a potential model for congenital cataract research.

## 1. Introduction

**Figure 1:**
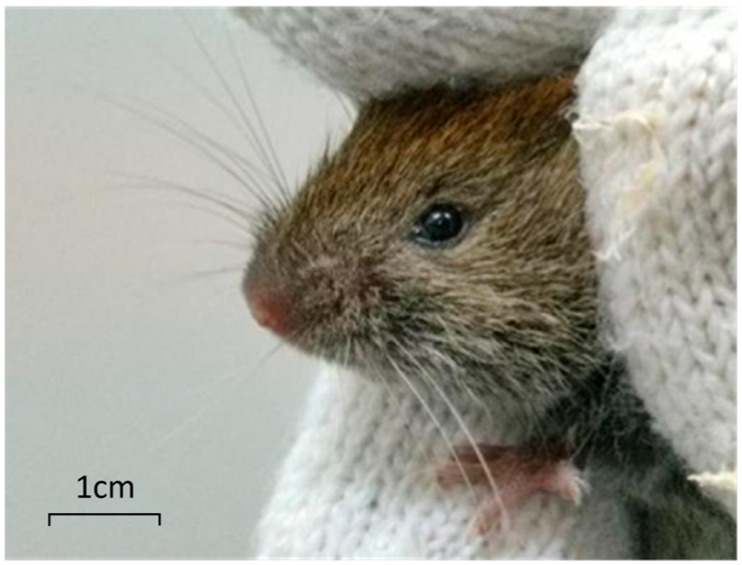
Normal eyeball of *M. rufocanus*.

**Figure 2:**
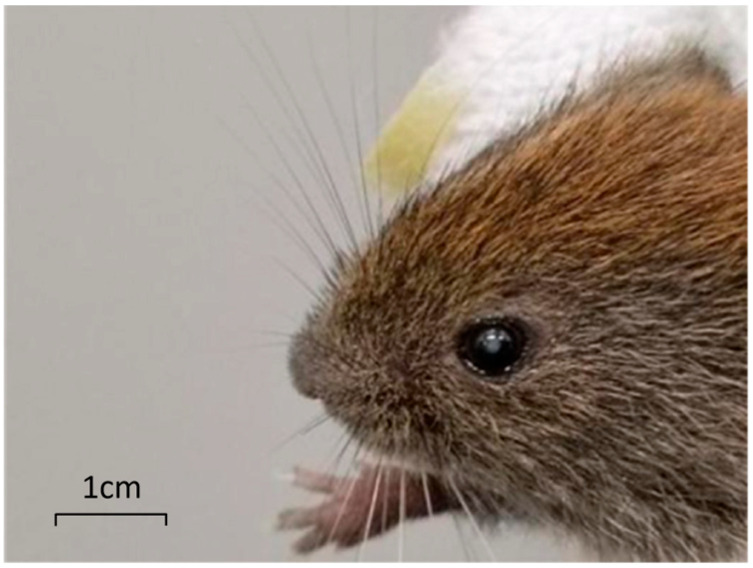
Cataract-affected eyeball of *M. rufocanus*.

Cataract is currently the leading cause of blindness worldwide, with over 15 million cataract patients as of 2020, accounting for 45% of 36 million blindness cases worldwide [1,2,3]. In the early stages of cataract disease, cataract has little effect on vision, but if not actively treated, it will gradually worsen, resulting in blurred vision; if not treated, it may even lead to blindness. The subfamily, mainly distributed in the northern forest areas of Hebei, Shanxi and Xinjiang, is the dominant species in northeast China. At present, most of the studies on *M. rufocanus* focus on the control of rodents and the pathogens they carry, mainly to control its population and reduce the spread of the epidemic. There is no relevant study on its own characteristics [4]. We found the congenital cataract phenotype when cultivating *M. rufocanus* (Figure 1 and Figure 2).

Although various animal models for congenital cataracts exist—including spontaneous mutants, genetically engineered lines, chemically induced models, and zebrafish models—each has limitations. Genetically engineered models rely on artificial intervention and cannot fully recapitulate the natural disease process. Existing spontaneous models are largely derived from long-term inbred mouse and rat strains with homogeneous genetic backgrounds. In contrast, the naturally occurring cataracts in *M. rufocanus* offer several unique advantages: (i) the phenotype arises spontaneously without any artificial induction, closely mimicking the natural course of human congenital cataracts; (ii) as a species of the Cricetidae family (Arvicolinae), it fills a phylogenetic gap, providing comparative insights into lens development and disease; and (iii) the closed colony maintains a relatively diverse genetic background, more akin to human populations, facilitating the identification of non-classical or low-frequency variants.

There are few studies at the genomic level in *M. rufocanus*, and no transcriptome sequencing results have been reported except for its mitochondrial genome [5]. Therefore, we performed reference-free transcriptome sequencing and compared gene expression differences between normal and cataractous eyes. In this study, we conducted the first transcriptome sequencing of *M. rufocanus* to analyze cataract-associated genes and their regulatory mechanisms, aiming to determine whether the observed phenotype represents congenital cataracts at the molecular level and to provide a reference for future studies on this species. We used RNA-seq to detect differentially expressed genes between normal and cataractous eyes, followed by functional enrichment analysis. The resulting datasets serve as important resources to facilitate the development of related research in *M. rufocanus*.

## 2. Materials and Methods

Experimental animals

*M. rufocanus* was the offspring of an indoor closed group of the changbai subspecies, which was introduced by Dalian Medical University and raised in the Experimental Animal Center of Jilin University (SYXK (Ji) 2020-0004). The feeding conditions were as follows: temperature maintained at 20~23 °C, humidity at 40~70%, a 12 h day and night cycle, and free feeding and drinking water. This study was approved by the Experimental Animal Ethics Committee of Jilin University (approval number: SY202302032).

Experimental grouping, sample collection and processing

Three 8-week-old male individuals with confirmed congenital cataracts (diagnosed at 4 weeks by gross observation and confirmed by slit lamp examination at 8 weeks) and three age-matched normal males (cataract group) were randomly selected, with a body mass of 20 to 30 g. Animals were randomly assigned to normal and cataract groups based on phenotype status.

Blood samples were obtained by orbital venous plexus blood sampling, part of which was placed in an EDTA-K2 anticoagulant tube for routine blood testing immediately, and part was placed at room temperature after centrifugation at 3000 r/min for 10 min and serum was collected for blood biochemical tests. Unilateral ocular tissue was removed for pathological sections and the other side was used for RT-PCR analysis. *M. rufocanus* were euthanized by carbon dioxide, and the carcasses were treated innocuously.

Slit lamp observation

*M. rufocanus* were anesthetized with 1.25% Avertin (0.2 mL/10 g, MA0478-1, meilunbio, Dalian, China) and then dilated with compound tropicamide eye drops (Xingqi, Shenyang, China), 1 to 2 drops each time, under an ophthalmic slit lamp microscope (Xinghe, Beijing, China) to observe the lens turbidity.

Blood physiological and biochemical testing

The whole blood samples were immediately tested using a blood cell analyzer (Mindray, Shenzhen, China), and the plasma samples were tested using an automatic blood biochemical analyzer (Mindray, Shenzhen, China), with the relevant reagents used as supporting products. The test indicators included white blood cell count (WBC), neutrophil count (Neu), lymphocyte count (Lym), monocyte count (Mon), eosinophil count (Eos), and basophil count (Bas); red blood cell parameters include red blood cell count (RBC), hemoglobin concentration (HGB), hematocrit (HCT), mean red blood cell volume (MCV), mean red blood cell hemoglobin content (MCH), and mean red blood cell hemoglobin concentration (MCHC); platelet parameters include platelet count (PLT), mean platelet volume (MPV), platelet volume distribution width (PDW), and platelet specific volume (PCT). Comparisons between the two groups were performed using an independent samples *t*-test, and a *p*-value < 0.05 was considered statistically significant.

Pathological histological analysis

Tissues were fixed in FAS fixative (Servicebio, Wuhan, China) for 24 h, dehydrated in graded ethanol, and embedded in paraffin. Sections (4 μm) were deparaffinized, rehydrated, stained with hematoxylin for 5 min, differentiated in acid alcohol, blued in tap water, counterstained with eosin for 2 min, dehydrated, cleared in xylene, and mounted with neutral balsam.

Transcriptome analysis

After grinding eyeball tissue, RNA was extracted using Trinzol method, and RNA was purified for cDNA synthesis, PCR amplification, purification and sequencing. Sequencing was performed on an Illumina HiSeq platform with paired-end 150 bp reads, generating 6.7–7.4 Gb of clean data per sample (Q30 > 93.5%).

De novo assembly and quality assessment: Clean reads from all samples were pooled and assembled using Trinity v2.4.0. The assembly quality was evaluated by calculating the N50 of transcripts and unigenes. Additionally, clean reads from each sample were aligned back to the assembled transcripts using Bowtie2 (v2.3.2) to obtain the mapping rate. Read duplication and gene coverage were assessed using RSeQC and BEDTools, respectively.

Analysis after sequencing: (1) RNA-seq sequencing evaluation: all obtained sequences were compared with the CDD, KOG, COG, NR, NT, PFAM, GONR databases using NCBI Blast+ (2.16.0), and the KAAS and KEGG automatic annotation servers were compared with the KEGG database to obtain the corresponding annotation information. The CDS prediction was performed using the Trans Decoder (v5.7.0). (2) Gene expression analysis: Bowtie2 was used to match the valid data of samples with spliced transcripts, and statistical mapping information. Based on the matching results, redundant sequence analysis was performed using RSeQC (5.0.4). Homogeneity distribution checks and gene coverage statistical analysis were performed using BEDTools (2.31.1). (3) Gene expression analysis: Salmon was used to calculate gene expression levels. Gene co-expression analysis was performed using WGCNA (1.73). Multidirectional statistical analysis and explorations were conducted, such as sample contrast analysis based on the sample expression matrix. (4) Differential expression analysis: gene expression differential analysis was performed, and the results of expression difference analysis were visualized. Wein plots and heatmaps were plotted and cluster analysis was performed based on the results of the difference analysis.

Quantitative fluorescence analysis of the expression of cataract-related genes in the eyeball

RNA samples to cDNA using a reverse transcription kit (Tiangen, Beijing, China).

The fluorescence quantification experiments were performed using the SuperReal PreMix Plus (SYBR Green) kit (Tiangen, Beijing, China) (reaction conditions: 94 °C, 3 min; 94 °C, 30 s; 60 °C, 30 s; a total of 40 cycles). The dissolution curves were examined after the end of the cycle, and the relative quantification of the target gene expression level was calculated using the 2-ΔΔCT method. Primer design for this experiment was performed by NCBI BLAST, and the specificity of the primers was tested using melting curves. Primer sequences: (GAPDH: F: TGAACGGGAAGCTCACTGGC, R: CATGTGAGATCCACGACGGACA; CRYBB2: F: ACCTGCTGGAGAAGGGAGAT, R: TCTAGCTGGAGGGGTGGAAG).

## 3. Results

### 3.1. Lens Changes in M. rufocanus Under Natural Light and Slit Lamp

**Figure 3:**
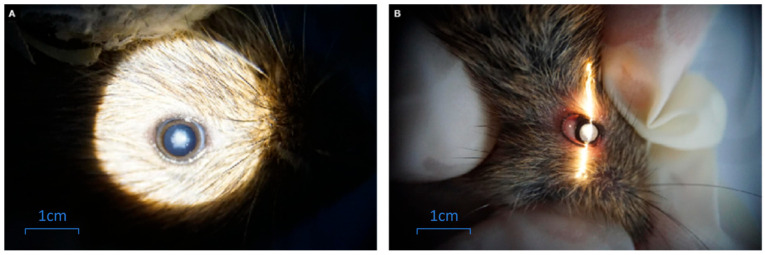
Examination of cataract by slit lamp: (**A**) normal eyeball; (**B**) cataract-affected eyeball.

We used slit lamps to examine *M. rufocanus* with a turbid phenotype to confirmed cataracts (Figure 3), and conducted further research accordingly.

### 3.2. Physiological and Biochemical Testing of M. rufocanus Blood

The blood cell count (WBC), lymphocyte count (Lym), and monocyte count (Mon) were significantly lower in the brown cataract group compared with the normal group, with no significant differences in other indicators (Table 1).

The blood glucose values in the control group and cataract group were 3.17 mmol/L, without any significant differences in other blood biochemical indexes, suggesting that *M. rufocanus* cataracts were not caused by metabolic diseases such as diabetes (Table 2).

### 3.3. M. rufocanus Histopathological Changes

**Figure 4:**
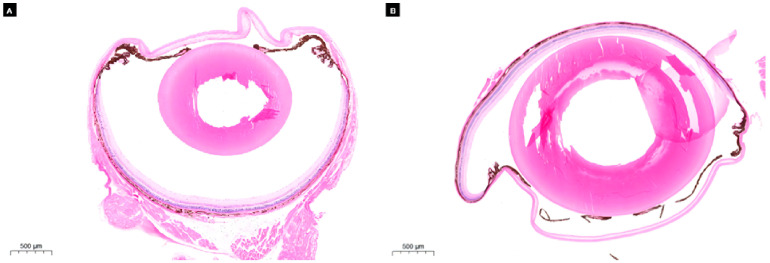
Sections of eye tissue from brown-backed mouse: (**A**) shows the normal brown-backed mouse eye (normal), and (**B**) shows the cataract eye (cataract).

We performed histological tests on normal *M. rufocanus* and cataract-affected the eyeballs of brown-backed mice respectively, and the HE staining results were as follows (Figure 4). Compared with normal *M. rufocanus*, the cataract group was severely damaged, and the eye lens structure had obvious eosinophilic aggregation in the core area. Control epithelial cells of the eye lens of a brown-backed mouse were ordered and intercellular structures were closely packed.

### 3.4. Transcriptome Results Analysis of the Brown-Backed Mouse Eye

We performed transcriptome sequencing of the brown-backed mouse eye in combination with the NCBI isogene database for gene annotation, but still nearly 50% of them were missing.

The de novo assembly yielded 37,564 transcripts with an N50 of 1409 bp and 21,480 unigenes with an N50 of 1058 bp. The mapping rates of clean reads to the assembled transcripts ranged from 83.5% to 92.0% across the three samples (LE: 92.0%, RE: 84.2%, CB: 83.5%), indicating that the assembly adequately represents the expressed transcriptome.

Meanwhile, through SSR analysis, we found a high proportion of simple repeats in gene sequences, with a SSR of 150.54/Mbp and a SSR of double base repeats of 140.82/Mbp.

Then, we screened DEGs and found that 6544 genes showed different expression, of which 3439 were upregulated and 3102 were downregulated. The annotation of gene functions is still incomplete due to the lack of studies on brown-backed mice. Therefore, in reference to studies of other species, we selected genes in DEGs for labeling, and identified some important genes associated with cataracts (Table 3). These include the *CRYBA4*, *CRYBB2*, and *CRYBB3* genes, which regulate lens proteins and transcription factors and are known to be involved in cataract pathogenesis.

**Figure 5:**
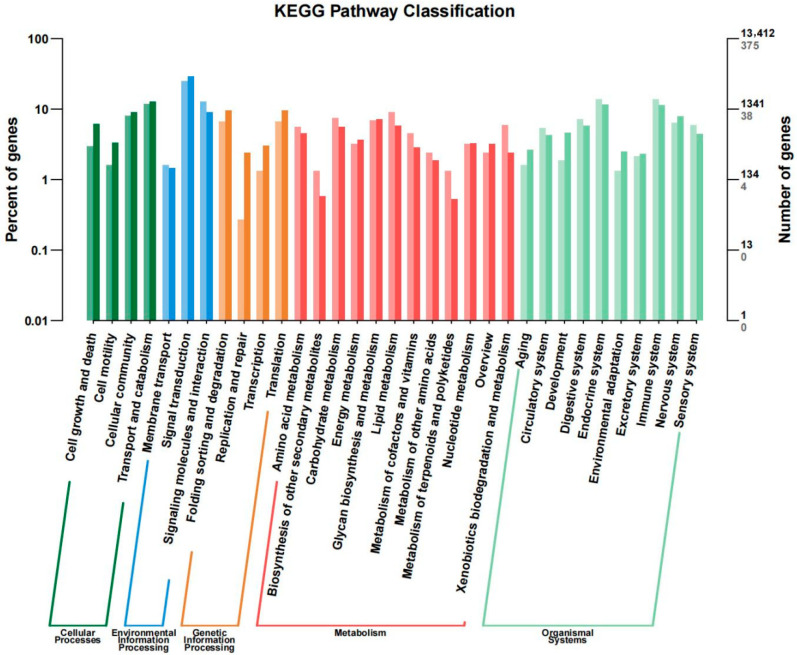
Differential analysis of KEGG pathway between normal and cataract groups.

KEGG libraries are commonly used for gene function analysis. We found that these DEGs are associated with signaling pathways including retinol metabolism, tyrosine metabolism, drug metabolism—cytochrome P450, and cytochrome P450-mediated metabolism of xenobiotics. We performed the analysis using DESeq. To obtain genes with significant differences, we set the screening condition to qValue < 0.05 and differential fold |log2FoldChange| ≥ 1. KEGG pathway results for DEGs were classified according to pathway type (as shown in Figure 5).

**Figure 6:**
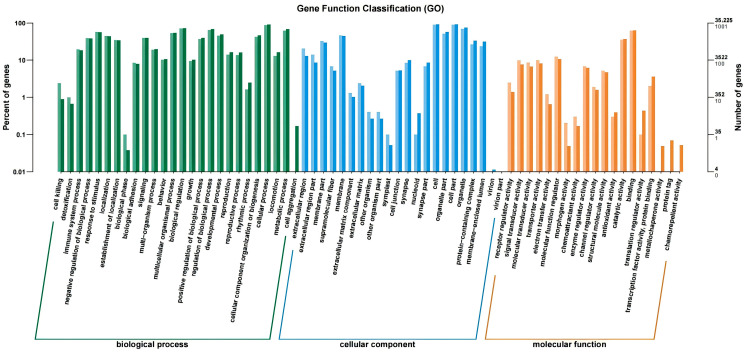
Gene function analysis between normal and cataract groups.

These genes lack annotation due to a previous lack of studies on gene function in brown-backed mice. Based on the differential expression results, we selected the 30 genes with the highest expression in cataract and normal eyes. The functions of these genes were classified based on studies in other species (Figure 6).

### 3.5. Expression of Cataract Disease-Causing Genes in the Brown-Backed Mouse Lens

**Figure 7:**
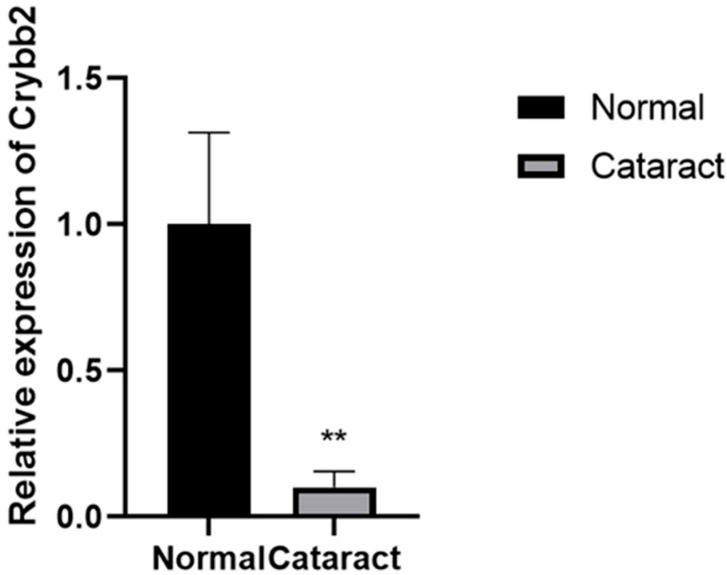
*CRYBB2* gene expression between normal and cataract groups. ** (*p* < 0.01).

Because there is no whole genome sequence of *M. rufocanus*, primers could not be designed directly. Sequences from closely related hamster species were selected for the primer design; specifically, *M. rufocanus Crybb2*. Compared with the control group, the cataract group *Myodes rufocanus* β lens protein (*CRYBB2*) gene expression significantly reduced (Figure 7).

## 4. Discussion

Current studies on *M. rufocanus* focus on the following aspects: taxonomic studies, feeding analysis, pathogen carriage studies and biological control, most of which are in the field of ecology and pathogen transmission [5,6,7].

In a taxonomic study, six unique cytochrome B number sequences isolated from brown-backed mice mitochondria were analyzed [8]. studied brown- and red-backed voles that share the same chromosome number and karyotype with little difference in chromosome composition and G-band type. The Ag-NORs of these mice are mitotic caps (CMC), and 10 autosomal pairs carry Ag-NOR in the *M. rufocanus* MRU [8]. In feeding research, Pierre et al. used a novel plant mixture DNA as a barcoding technique and a traditional microhistorical method to understand food composition and food preferences in the stomachs of herbivores [7].

In the present study, the cataract group showed significantly lower white blood cell (WBC) and lymphocyte (Lym) counts compared with the normal group (Table 1). The underlying cause of this reduction remains unclear at this stage. One possible explanation is that the presence of congenital cataract may be associated with a low-grade chronic inflammatory state or altered immune homeostasis. Chronic lens opacity could induce local tissue stress or microdamage, potentially triggering systemic immune modulation. Alternatively, the observed differences might reflect nutritional, metabolic, or stress-related factors inherent to the affected individuals. However, given the small sample size and the lack of additional immunological parameters (e.g., cytokine profiles, lymphocyte subpopulations), this finding should be interpreted with caution. Further studies incorporating immune function assays are needed to determine whether the leukocyte changes are directly linked to the cataract phenotype or represent incidental variations.

Returning to the molecular basis of cataracts, the pathogenesis of congenital cataracts has received close attention; however, it is still unclear, and various genetic mutations and mutation patterns may lead to congenital cataracts. Based on the established literature, Pitx3 Gene mutations can cause congenital cataracts in the early stages of lens development [9]. Genes causing abnormal lens structure, such as the *CRYBB2* gene encoding the β-lens protein, include a common mutation called c.463C>T(p.Q155X) [10] can cause cortical, nuclear, and total cataract opacity types [11]. Therefore, eyes with cataract and normal eyes were selected as samples for comparative analysis to provide a reference for further finding whether congenital cataracts could allow further study of *M. rufocanus*.

There is no reference sequence for *M. rufocanus* transcriptome sequencing, so we chose to conduct reference-free transcriptome sequencing analysis, selected three samples from this family, and performed transcriptome sequencing with three normal *M. rufocanus* families for further analysis. Since no transcriptome analysis was used, no additional data could be provided for analyzing interindividual differences. It is hoped that the data from the subsequent study can provide a reference and supplement to this study.

Differences in gene expression have been shown to play an important role in individual life activities [12,13], and transcriptomic studies in humans, pigs, mice and rats have shown significant differences in gene expression between different tissues in the same body [14,15]. The RNA-Seq maps of gene expression in normal mouse and rat tissues were constructed by Sollner et al. Comparative genomics has shown that many genes with conserved sequences also show highly correlated tissue distribution. Gluck et al. identified changes in transcriptomic regulation of different cell populations during development and differentiation in mouse salivary glands by RNA-seq [16]. Therefore, by comparing the transcriptome of different samples to explore whether this *M. rufocanus* population had congenital cataracts, this study collected and sequenced the cataract and normal eyes and established a database to provide qualified RNA that met the quality standards of the Illumina platform. The proportion of Q30 bases in all samples was not lower than 96.38%, indicating sufficient quality for subsequent experimental analysis.

In this study, we investigated the transcriptome of normal brown-backed mice eyes and cataract-affected eyeballs using RNA-seq technology. Cluster analysis showed that DEGs were mainly related with retinol metabolism, tyrosine metabolism, drug metabolism—cytochrome P450, cytochrome P450 and cytochrome P450 metabolism. The lack of a reference genome for *M. rufocanus* resulted in ~48% of unigenes lacking annotation, limiting the interpretation of a substantial portion of DEGs. Among the annotated genes, the enriched KEGG pathways (retinol metabolism, tyrosine metabolism, cytochrome P450) are known to regulate lens differentiation and oxidative stress, and their disruption can contribute to cataractogenesis. Importantly, the downregulation of crystallin genes (*CRYBB2*, *CRYBA4*, *CRYGS*) in our cataract group is consistent with the established mechanisms, as reduced crystallin expression leads to protein aggregation and lens opacity in humans and other animal models. These findings suggest that the *M. rufocanus* cataracts shares molecular features with human congenital cataracts. Functional annotation of DEGs indicate that expression levels of cataract-associated pathogenic genes are downregulated in cataract-affected brown-backed mouse eyes.

Before discussing our findings, it is important to note the following well-established associations from previous studies (known mechanisms): *CRYBA1* (c.272delGAG; Loss of p.G91del) is associated with nuclear cataract, splice variant of *CRYBA2* (c.446 + 1G>A), *CRYBA4* (c.225G>T) [17] the *CRYBB3* missense variant c.165G>C (p.Gly3Arg) [18], and *CRYGS*, the missense mutation c.199T>A, the p. (Tyr67Asn) [19] are associated with congenital cataracts. Mutations in GJA8 NM_005267.5:c.124G>A, p. (E42K) cause congenital nuclear cataracts [20], GJA3 (c.188A>G, p. (Asn3Ser)) can cause congenital nuclear and cortical cataracts [21], and FOXE3-p.Ala170Ala (rs34082359) and PITX3-p.Ile95Ile (rs2281983) polymorphisms are associated with congenital cataracts. Mutations in the *HSF4* gene are mainly associated with congenital and early-onset cataracts, and mutations with an autosomal dominant and recessive mode of inheritance [22] Mutations in the Maf auxiliary DNA binding domain (R288P) have been shown to cause cataracts [23]. In our sample, as a preliminary observation, the expression levels of these causative genes associated with congenital cataracts were all downregulated in the cataract-affected eyeball, suggesting that these genes may be associated with this cataract phenotype.

Further known pathogenic mechanisms of congenital cataracts include the following: for example, mutations in the Pax 6 gene can cause subcapsular cataracts, anophthalmia [24], anterior and posterior pole cataracts in the early lens development stage [24]. The *CRYBB2* gene encoding the β-lens protein causes abnormal lens structure; the most common mutation is c.463C>T(p.Q155X), which can cause cortical cataracts, nuclear cataracts, and total cataracts. And also the *MIP* gene mutation c.572C>G results in reduced expression of AQP 0 on the surface of cell membranes, allowing abnormal water molecules in and out of cells, and abnormal lens metabolism, resulting in total lens cataract [25]. Genes that regulate the crystalline lens development, like the c. 81 hhh 2 T>A mutation in the EHR region of the *Maf* gene, affect oxidative stress in the lens by limiting the expression of target genes. This disease presents as a nuclear cataract [26].

To further explore the pathogenic genes and mechanism, genes listed in Table 1 were selected for RT-PCR; because there is no *M. rufocanus* genome sequence, primers were designed based on the golden mouse gene sequence for experiment, and we found that gene expression of the *CRYBB2* gene in cataract-affected *M. rufocanus* was down. Based on these preliminary results, we speculate that *CRYBB2* gene may be related to *M. rufocanus* cataract expression regulation; however, this remains speculative, limited by the current lack of whole genome sequencing results, and further exploration of the relevant pathogenic genes and mechanism remains to be carried out. In addition, several limitations of this study should be acknowledged.

In the present study, only three individuals per group were included. This is because although we have successfully established a colony of *M. rufocanus* with a stably heritable congenital cataract phenotype, the low reproductive efficiency of this species (e.g., small litter size, relatively long maturation period) precluded the acquisition of a larger number of eligible affected individuals within a reasonable timeframe. Therefore, this study should be considered a pilot investigation providing preliminary descriptive data on the cataract phenotype and transcriptomic profile of this species. The statistical comparisons (e.g., blood parameters, differentially expressed genes) are based on this limited sample size, and the results should be interpreted with caution. Future studies with larger cohorts are necessary to validate the observed differences, confirm the heritability of the cataract phenotype, and further elucidate the underlying molecular mechanisms. Nevertheless, despite the small sample size, the consistent histopathological changes and the downregulation of crystallin genes (e.g., *CRYBB2*, *CRYBA4*, *CRYGS*) across all three cataract individuals support the robustness of the main findings.

## 5. Conclusions

Through the pathological, physiological, biochemical and genetic analyses of the *M. rufocanus*, this pilot study provides preliminary evidence that *M. rufocanus* with naturally occurring cataracts may serve as a potential animal model, but further validation with larger sample sizes and heritability studies is required.

## Figures and Tables

**Table 1:** Analysis of physiological indexes of *M. rufocanus* blood in normal and cataract groups.

	Normal Group	Cataract Group
WBC (10^9^/L)	1.82 ± 0.13	1.09 ± 0.02
Neu # (10^9^/L)	0.18 ± 0.06	0.18 ± 0.03
Lym # (10^9^/L)	1.46 ± 0.19	0.78 ± 0.08
Mon # (10^9^/L)	0.12 ± 0.05	0.1 ± 0.04
Eos # (10^9^/L)	0.06 ± 0.01	0.03 ± 0.03
Bas # (10^9^/L)	0 ± 0.00	0 ± 0
Neu % (%)	9.7 ± 3.41	5.9 ± 2.78
Lym % (%)	79.9 ± 5.70	70.9 ± 7.97
Mon % (%)	6.87 ± 3.10	9.63 ± 4.01
Eos % (%)	3.33 ± 0.67	3.5 ± 3.00
Bas % (%)	0.2 ± 0.35	0.07 ± 0.12
RBC (10^12^/L)	9.66 ± 1.21	9.21 ± 1.64
HGB (g/L)	153 ± 17.58	147 ± 35.00
HCT (%)	39.23 ± 5.04	39.47 ± 7.82
MCV (fL)	40.63 ± 1.94	42.8 ± 1.18
MCH (pg)	15.83 ± 0.21	15.83 ± 0.87
MCHC (g/L)	390.67 ± 22.05	370 ± 18.73
RDW-CV (%)	12.9 ± 0.79	14.23 ± 0.75
RDW-SD (fL)	22.7 ± 2.69	26.57 ± 0.32
PLT (10^9^/L)	409 ± 76.21	447.67 ± 122.32
MPV (fL)	4.9 ± 0.75	4.8 ± 0.61
PDW (%)	15.77 ± 0.21	15.5 ± 0.35
PCT (%)	0.2 ± 0.05	0.22 ± 0.08

**Table 2:** Analysis of biochemical indexes of *M. rufocanus* blood in normal and cataract groups.

	Normal Group	Cataract Group
ALT	116.47 ± 2.79	111.40 ± 9.62
AST	326.17 ± 36.00	326.7 ± 16.87
ALB	42.8 ± 2.08	42.07 ± 6.57
UREA	6.22 ± 0.22	7.72 ± 1.15
CREA-S	26.47 ± 7.30	24.83 ± 1.93
GLU-G	3.17 ± 0.51	3.17 ± 0.51
TP2	61.3 ± 1.74	59.00 ± 8.42
TC	2.5 ± 0.46	2.15 ± 0.47
TG	0.73 ± 0.20	0.55 ± 0.21

**Table 3:** Genes associated with congenital cataracts among the genes significantly different between normal and cataract groups.

Gene	Gene id	MeanTPM (T)	MeanTPM (C)	log2FoldChange	*p* Value	q Value	Result
*CRYBA4*	TRINITY_DN1059_83_c0_g1	4.831032667	10.9069265	−1.174841104	6.24159 × 10^−7^	6.93998 × 10^−5^	down
*CRYBB2*	TRINITY_DN12097	0.661233833	3.832961667	−2.53522712	9.43316 × 10^−8^	1.38287 × 10^−5^	down
*CRYBB3*	TRINITY_DN11253	7.652753667	28.19613833	−1.881446722	0.000155279	0.006090819	down
	TRINITY_DN11340	299.0457839	1003.509257	−1.746615643	3.70015 × 10^−5^	0.002024292	down
*CRYGS*	TRINITY_DN11253	744.6663483	3103.17669	−2.059079776	0.002696894	0.048259947	down

## Data Availability

Data available on request from the authors.
